# X-Linked Retinitis Pigmentosa Caused by Non-Canonical Splice Site Variants in *RPGR*

**DOI:** 10.3390/ijms22020850

**Published:** 2021-01-16

**Authors:** Friederike Kortüm, Sinja Kieninger, Pascale Mazzola, Susanne Kohl, Bernd Wissinger, Holger Prokisch, Katarina Stingl, Nicole Weisschuh

**Affiliations:** 1 Center for Ophthalmology, University Eye Hospital, University of Tübingen, 72076 Tübingen, Germany; Friederike.Kortuem@med.uni-tuebingen.de (F.K.); Katarina.Stingl@med.uni-tuebingen.de (K.S.); 2 Center for Ophthalmology, Institute for Ophthalmic Research, University of Tübingen, 72076 Tübingen, Germany; sinja.kieninger@med.uni-tuebingen.de (S.K.); susanne.kohl@uni-tuebingen.de (S.K.); wissinger@uni-tuebingen.de (B.W.); 3 Institute of Medical Genetics and Applied Genomics, University of Tübingen, 72076 Tübingen, Germany; Pascale.Mazzola@med.uni-tuebingen.de; 4 Institute of Neurogenomics, Helmholtz Zentrum München, 85764 Neuherberg, Germany; prokisch@helmholtz-muenchen.de; 5 Institute of Human Genetics, Technische Universität München, 81675 Munich, Germany

**Keywords:** retinitis pigmentosa, X-linked, RPGR, non-canonical splice site variant, in vitro splice assay

## Abstract

We aimed to validate the effect of non-canonical splice site variants in the *RPGR* gene in five patients from four families diagnosed with retinitis pigmentosa. Four variants located in intron 2 (c.154 + 3_154 + 6del), intron 3 (c.247 + 5G>A), intron 7 (c.779-5T>G), and intron 13 (c.1573-12A>G), respectively, were analyzed by means of in vitro splice assays. Splicing analysis revealed different aberrant splicing events, including exon skipping and intronic nucleotide addition, which are predicted to lead either to an in-frame deletion affecting relevant protein domains or to a frameshift of the open reading frame. Our data expand the landscape of pathogenic variants in *RPGR*, thereby increasing the genetic diagnostic rate in retinitis pigmentosa and allowing patients harboring the analyzed variants to be enrolled in clinical trials.

## 1. Introduction

Retinitis pigmentosa (RP) encompasses a heterogeneous group of hereditary, primarily monogenetic retinal diseases characterized by a primary degeneration of rod and secondary degeneration of cone photoreceptors. RP occurs with a frequency of 1:4000, and affects more than 1 million people world-wide [1]. Clinical hallmarks of RP include bone spicule pigmentation, attenuated retinal vessels, optic disc pallor, visual field loss, and reduced or non-recordable responses in electroretinography (ERG) as well as pathological dark adaption. Non-syndromic RP is most commonly inherited in an autosomal-recessive manner (autosomal dominant retinitis pigmentosa (ARRP), 50%–60%), followed by an autosomal-dominant (autosomal dominant retinitis pigmentosa (ADRP), 30%–40%) and X-linked (X-linked retinitis pigmentosa (XRP), 5%–15%) pattern [2,3]. Small core families can hamper the assignment of the mode of inheritance. Hence, single patients are classified as simplex or sporadic RP (SRP). The rate of disease progression is highly variable between the different types of RP. XRP is a particularly severe and early onset form of RP predominantly affecting males, although female carriers can be symptomatic due to unfavorably skewed X-chromosome inactivation [4,5,6]. The most common cause of XRP are pathogenic variants in the retinitis pigmentosa GTPase regulator (*RPGR*) gene (MIM#312610). Depending on cohort composition and analysis technique, 70%–90% of XRP patients are found to harbor causative variants in *RPGR* [7]. While there is no effective cure for *RPGR*-associated XRP, a recent gene augmentation therapy trial has raised new hopes by demonstrating significant vision improvement in treated patients [8]. *RPGR* encodes a protein which is localized to the base of the connecting cilium of the photoreceptor and thought to function in microtubule organization and ciliary protein trafficking [9]. The exact function of RPGR is yet unclear. The N-terminal region of RPGR shows homology to the regulator of chromosome condensation 1 (RCC1), which serves as a guanine nucleotide exchange factor for a GTPase involved in nucleocytoplasmic transport [10]. To date, the corresponding GTP-binding protein has not been identified for RPGR. Transcript studies have demonstrated complex alternative splicing patterns for the *RPGR* gene, resulting in a multitude of transcripts [11,12,13]. The constitutive isoform termed *RPGR*^Ex1‑19^ is expressed widely throughout the body, while the predominant isoform of the retina is termed *RPGR*^ORF15^ and utilizes a large open reading frame (ORF15) as C-terminal exon [14,15]. All mutations known to be disease-causing occur in the *RPGR*^ORF15^ isoform [14,16]. As of November 2020, the Human Gene Mutation Database (HGMD) lists more than 500 different *RPGR* mutations linked to RP, cone-rod, cone, and macular dystrophies, or syndromic forms of XRP. The majority of the mutations are nonsense and frameshift variants, while roughly 10% are splice variants. Of these, approximately one third is located in the vicinity but outside the highly conserved GT and AG splice acceptor and splice donor dinucleotides. Such non-canonical splice site (NCSS) variants are less straightforward to interpret since they exhibit considerable sequence variation. Hence, according to the standards and guidelines provided by the American College of Medical Genetics and Genomics (ACMG), NCSS variants have to be considered as variants of uncertain significance (VUS) unless their effect on splicing has been validated [17]. Direct analysis of native transcripts would be the most reliable approach but is hampered by the retina-specific expression of the *RPGR*^ORF15^ isoform. In this study, we aimed to validate the consequences of four NCSS variants in *RPGR* using in vitro splice assays.

## 2. Results

### 2.1. Clinical Phenotype

**Figure 1:**
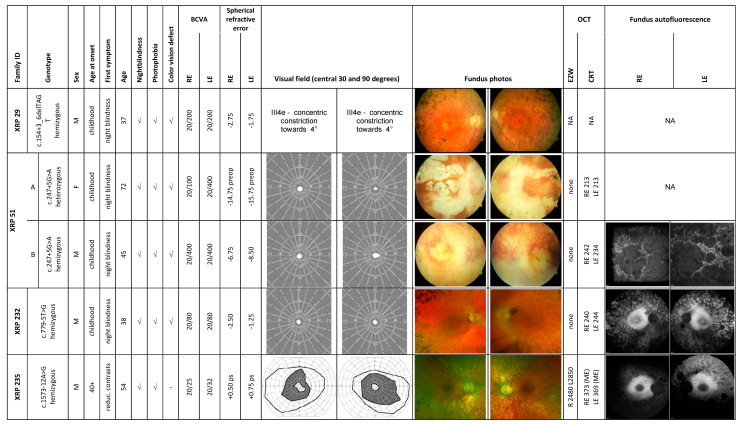
Summary of clinical findings. The composite image shows the most important available clinical findings. Each row represents a single patient with the following information in each column: Family ID; genotype; gender; age of onset (as reported subjectively by the patient); first symptom (first manifestation of the disease as reported by the patient); age; night blindness, photophobia, and color vision defect (“✓” for present, “-” for absent); best corrected visual acuity (BCVA); spherical refractive error; visual field; fundus image; ellipsoid zone width (EZW) in µm; central retinal thickness (CRT) in µm; fundus autofluorescence image (if available). RE, right eye; LE, left eye; F, female; M, male; ME, macular edema; NA, not available; ps, pseudophakia; and grey area in visual field indicates absolute scotoma.

Figure 1 provides an overview of patients’ demographics, visual complaints and ophthalmological examination findings, including best corrected visual acuity (BCVA), typical RP symptoms (night blindness, photophobia, and color vision defects), onset of RP symptoms, perimetry, fundus images, fundus autofluorescence images (FAF), and optical coherence tomography (OCT) findings.

Patient ages range from 37 to 72 years. Four patients were male and one was female (XRP 51 A). BCVA ranged from 20/400 (both members of family XRP 51) to 20/25 (XRP 235). All patients except XRP 235 reported night blindness during childhood as the first symptom. Color vision disturbances were reported by all patients with the exception of XRP 235. Farnsworth Panel D15 testing showed profound color vision defects in XRP 29 and XRP 51 (data not shown) while color vision testing was not available for patients XRP 232 and XRP 235. Scotopic and photopic ERGs were not recordable in any patient with the exception of patient XRP 235 who showed residual photopic ERG responses (data not shown). Perimetry revealed severe concentric constriction of the visual fields under 5 degrees for all patients except for patient XRP 235. For this patient, a wide ring scotoma was found with preservation of the peripheral visual field. It is not known at which age the perimetry reached a severe level of constriction under 5 degrees as we only examined the patients during adulthood. OCT scans revealed no discernable ellipsoid zone (*EZ*) band except for patient XRP 235 (see Supplementary Materials Figure S1). This patient also presented with a macular edema at his last visit. Fundus examination revealed typical bone spicule-like pigment in the mid-periphery and periphery in all patients. Both members of family XRP 51 had high myopia fundus changes with chorioretinal atrophy. Of note, patient XRP 51 A, who is the mother of patient XRP 51 B, presented with a severe phenotype that is rather unusual for female carriers and is indicative of unfavorably skewed X-inactivation [4,5,6].

The observed phenotype was consistent with a diagnosis of RP in all patients. Of note, patient XRP 235 presented with a milder phenotype than the other four patients. This patient also showed a hyperfluorescent ring around the macula indicating the zone of photoreceptor loss.

### 2.2. In Silico Analysis

**Figure 2:**
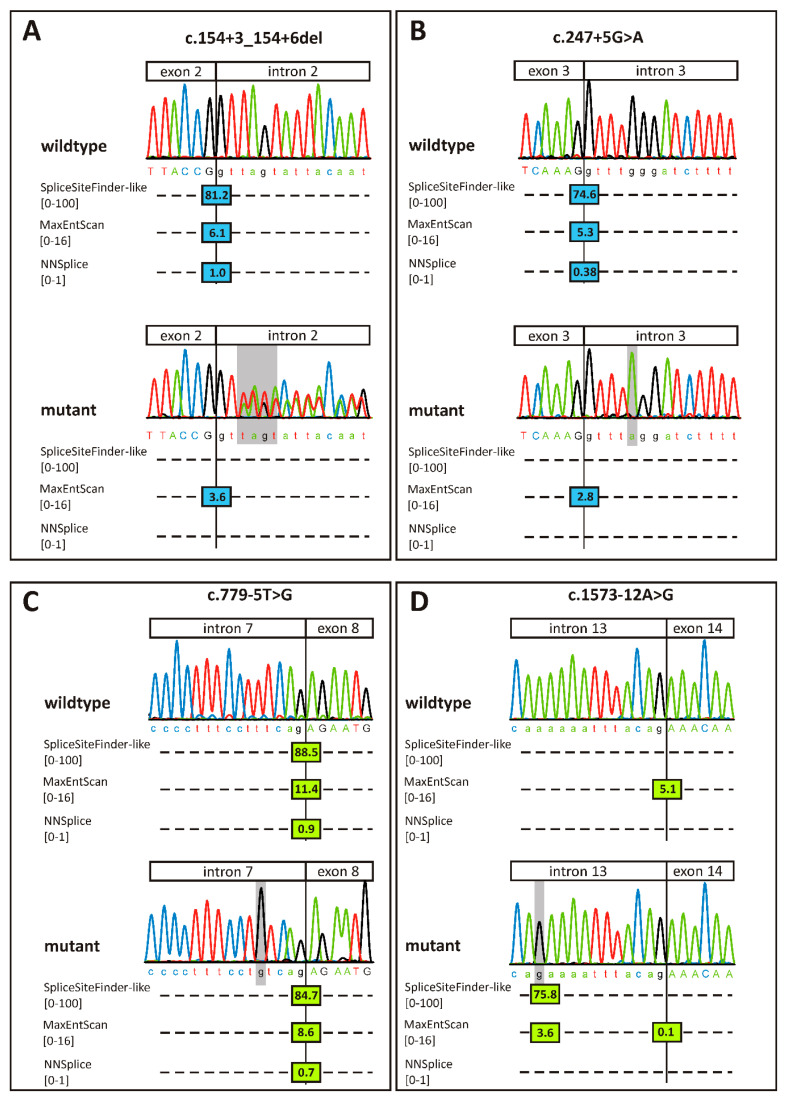
Splice site predictions for the four variants analyzed in this study. (**A**) c.154+3_154+6del, (**B**) c.247+5G>A, (**C**) c.779-5T>G, (**D**) c.1573-12A>G, Exonic nucleotides are given in capital letters, intronic nucleotides are given in lower case letters. Exon–intron borders are indicated with a vertical line. Variant nucleotides are highlighted with a gray bar. Donor site scores from three different algorithms embedded in the Alamut visual software (v.2.12) are displayed as blue vertical bars. Acceptor site scores are displayed as green bars. The respective prediction score is provided within each bar. Note that scores on the higher end of the given scales represent a higher probability of a splice site. The fourth algorithm of the Alamut software, namely GeneSplicer, was not included in the figure since it did not recognize any of the splice sites. Note that for variant c.154 + 3_154 + 6del the sequence of the patient’s mother (who is heterozygous for the variant) is depicted in order to better demonstrate the 4 bp deletion of the mutant allele. Wildtype sequences were derived from a healthy control person.

Diagnostic genetic testing of the patients in this study provided inconclusive results since the only putative pathogenic variants identified were NCSS variants in the *RPGR* gene with the exception of patient XRP 232 who also carries two putatively pathogenic missense variants in *USH2A*. None of the four *RPGR* variants was observed in the population database gnomAD (https://gnomad.broadinstitute.org/), indicating that they are very rare. While the rarity of an allele is widely recognized as an indicator of pathogenicity, it is not a sufficient criterion [17]. To evaluate the pathogenicity of the NCSS variants described in this study, we first performed an in silico analysis using different prediction programs (Figure 2). The wildtype donor site of exon 2 achieves high scores with three algorithms, but is predicted to be virtually abolished if disrupted by variant c.154 + 3_154 + 6del (Figure 2A). The same holds true for variant c.247 + 5G>A which affects the donor site of exon 3 (Figure 2B). In contrast, variant c.779-5T>G is predicted to only slightly weaken the acceptor site of exon 8 (Figure 2C) while two algorithms predict that variant c.1573-12A>G creates a novel acceptor site for exon 14 (Figure 2D).

### 2.3. In Vitro Splice Assays

Owing to the lack of *RPGR* expression in accessible patient tissues, we made use of heterologous splice assays (also often referred to as minigene assays) in human embryonic kidney (HEK)293T cells to test mutant and wildtype RPGR minigene constructs in direct comparison.

**Figure 3:**
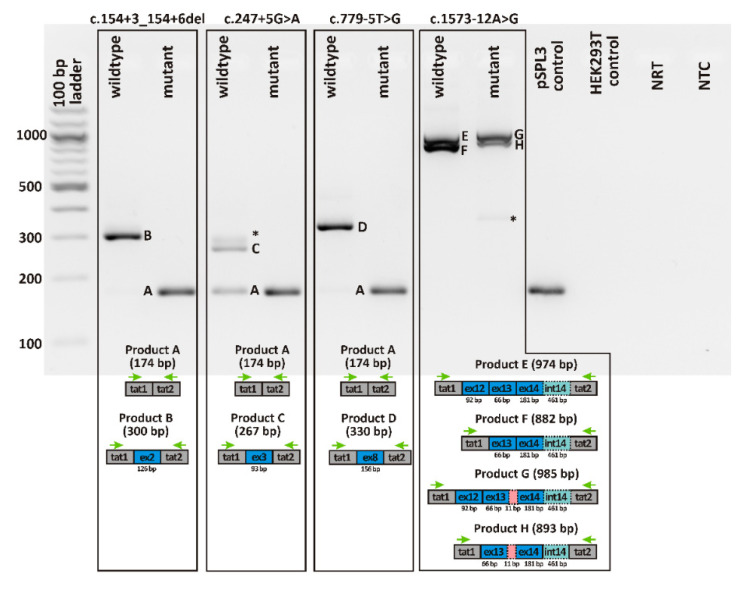
Qualitative analysis of RT-PCR products from in vitro splicing assays. Gel loading is as follows: A 100 bp ladder size standard is loaded in the leftmost lane. RT-PCR products derived from human embryonic kidney (HEK)293T cells transfected with plasmid constructs harboring the wildtype allele of the respective variant are shown in lanes 2, 4, 6, and 8, while those with the mutant allele are shown in lanes 3, 5, 7, and 9. RT-PCRs from transfection with empty pSPL3 vector (lane 10) and untransfected HEK293T cells (lane 11) served as controls. NRT (lane 12), no reverse transcriptase control; NTC (lane 13), no template control. Schemes of the amplified products are presented below the agarose gel. Grey boxes represent pSPL3 resident exons tat1 and tat2, and blue boxes retinitis pigmentosa GTPase regulator (RPGR) exons, respectively. Dotted lines represent retained intronic sequence. The green arrows indicate the location of the RT-PCR primers. Asterisks indicate PCR products that could not be captured by subcloning.

Figure 3 shows the reverse transcription polymerase chain reaction (RT-PCR) products obtained upon transfection of HEK293T cells with minigene constructs harboring either the respective wildtype or mutant allele of the four variants. RT-PCR was performed using primers binding to the pSPL3 resident exons tat1 and tat2. For variants c.154 + 3_154 + 6del, c.247 + 5G>A, and c.779-5T>G, transfection with the mutant construct yielded an RT–PCR product that was clearly smaller than the product obtained from cells transfected with the wildtype allele. Subsequent sequencing of RT-PCR products confirmed skipping of the entire exon for all three variants (Supplementary Materials Figure S2) and correct splicing for the wildtype alleles (i.e., the respective RPGR exon spliced between the tat1 and tat2 exons). Of note, exon skipping could also be observed for the respective wildtype allele for all three variants. The quantities were very low, and hardly visible on the agarose gel, in comparison to the correctly spliced transcript for variants c.154 + 3_154 + 6del and c.779-5G>T, but in case of variant c.247 + 5G>A reached amounts roughly equal to that of the correctly spliced product.

The splicing pattern for variant c.1573-12A>G was more complex than for the other three variants as two RT-PCR products were observed both for the wildtype and the mutant allele. Subcloning of RT-PCR products and subsequent sequencing of individual clones showed that the mutant allele leads to the retention of the last eleven nucleotides of intron 13. The smaller product observed for both the wildtype and the mutant allele corresponds to transcripts lacking exon 12 (Supplementary Figure S3).

## 3. Discussion

In this study, we describe the effect of four NCSS variants in the *RPGR* gene using in vitro splice assays and thereby confirm their pathogenicity. To the best of our knowledge, two of the variants analyzed in our study, namely c.247 + 5G>A and c.1573-12A>G, have not been described before. In contrast, variants c.154 + 3_154 + 6del and c.779-5T>G have already been published as the underlying cause of disease in two XRP patients. However, the consequence of the variants was not validated, and clinical details were not provided [18]. Very recently, variant c.779-5T>G was reported in another study and linked to a hypomorphic phenotype of XRP [19]. Splicing analysis of the variant based on a reporter construct showed a reduced efficiency of intron splicing compared with the wildtype [19].

Our minigene assays for variants c.154 + 3_154 + 6del, c.247 + 5G>A, and c.779-5T>G clearly demonstrate skipping of the respective exon in all three cases. The aberrant transcripts of all three variants would lead, if translated, to shortened proteins lacking important protein domains. To date, no GTP-binding protein has been identified that substantiates the hypothetical role of RPGR as a guanine nucleotide exchange factor. However, variant c.154 + 3_154 + 6del would preclude the binding of this orphan GTP-binding protein to RPGR as the translated protein (p.D10_T51del) is predicted to lack the two GTP phosphate binding sites at amino acid positions 13–19 (AVFTFGKS), and 31–36 (WFKNDV), respectively [20]. The predicted consequence of variant c.247+5G>A on amino acid level would be a protein that lacks the first 31 amino acids of the RCC1-like domain (p.G52_K82del), while variant c.779-5T>G would lead to a protein that lacks 52 amino acids within the RCC1-like domain (p.E260_T311del). The RCC1-like domain of RPGR has been shown to interact with the RPGR interacting protein 1 (RPGRIP1) and the phosphodiesterase 6 delta subunit (PDE6δ) [21,22]. As was shown previously, RPGR forms a seven-bladed β propeller [23]. The lack of 31 or 52 amino acid residues of the RCC1-like domain is likely to interfere with the formation of the β sheets. We hypothesize that variants c.247 + 5G>A and c.779-5T>G influence the binding of RPGRIP1 and PDE6δ to the RCC1-like domain. As mentioned above, variant c.779-5T>G has been analyzed in a previous study and linked to a hypomorphic phenotype of XRP [19]. The index patient in the previous study presented with good visual acuity at age 84 years and a fundus appearance that resembled choroideremia [19]. The phenotype of our patient XRP 232, who harbors the c.779-5T>G variant, is much more severe. Our patient is 38 years old and he was diagnosed more than 40 years earlier than the patient from the previous study. However, a drawback of our study is that the phenotype of patient XRP 232 cannot be univocally attributed to the c.779-5T>G variant in *RPGR* since he also carries two missense variants in *USH2A* (Supplementary Figure S4). Variant c.653T>A/p.V218E has been repeatedly described as pathogenic in RP and Usher syndrome [24,25,26,27], while variant c.9545A>G/p.H3182R is novel. Of note, patient XRP 232 did not complain about hearing problems but audiometry tests have not been performed to validate his subjective perception. According to the self-reported family history, the patient´s father suffered from RP and was legally blind in his forties. As the patient´s father was not available for genotyping, we could not univocally confirm that the two *USH2A* variants found in patient XRP 232 are in trans. However, analysis of the patient´s mother confirmed her to be a heterozygous carrier of both the *RPGR* c.779-5T>G allele and the *USH2A* c.653T>A allele. She reported to have problems with night vision but was not clinically examined. Notably, the maternal grandfather has been reported to have suffered from visual impairment, too. We hypothesize that two disease entities run in the pedigree, namely XRP caused by a pathogenic *RPGR* allele and inherited through the maternal line, and ARRP caused by two pathogenic *USH2A* alleles inherited through both the maternal and the paternal line. This may explain the much more severe phenotype of our patient when compared to the patient described in the aforementioned study [19].

With respect to the fourth variant analyzed in this study, our results demonstrate that the mutant allele of the c.1573-12A>G variant creates a novel acceptor site for exon 14. The aberrant transcript would lead, if translated, to an insertion of ten novel amino acid residues followed by a premature stop codon (PTC) (p.Q526Ifs*11). In *RPGR*, nonsense variants within exons 1–14, or frameshift mutations in exons 1–14 leading to PTCs, are predicted to result in nonsense mediated decay (NMD) of the transcript and are therefore considered to represent null alleles [28]. However, patient XRP 235, who harbors the c.1573-12A>G variant, shows the mildest phenotype of all four patients analyzed in this study. At age 56 his BCVA was still 20/25 and 20/32. Morphologically, OCT revealed a central island of relatively preserved retina. The comparably mild phenotype at age 56 is unusual for *RPGR*-associated RP [29,30], in particular for a variant that is predicted to represent a null allele [28]. In our minigene assay for the c.1573-12A>G variant, subcloning of vector-derived transcripts did not reveal correctly spliced transcripts for the mutant allele, indicating that the variant is fully penetrant. On the other hand, the comparably mild phenotype of patient XRP 235 suggests residual RPGR function. It has been shown that NMD efficiency can vary between different cell types [31]. In addition, while minigene splicing assays and patient RNA analyses are mostly concordant, qualitative differences in splicing products have been reported repeatedly [32,33,34,35]. Moreover, mechanisms like basal exon skipping or nonsense-associated altered splicing as described for another retinal disease gene, namely *CEP290*, may well explain these apparent contradictory findings [36,37].

Of note, the minigene constructs we used to analyze the effect of the c.1573-12A>G variant gave rise to two additional splicing events, namely the skipping of exon 12, and the retention of intron 14 (i.e., the first 461 nucleotides of intron 14 that were part of the minigene constructs). The presence of two products in the RT-PCR necessitated subcloning. Sequencing of individual subclones revealed that all of them showed intron 14 retention while approximately 50% showed exon 12 skipping. Neither of these splicing events can be linked to the c.1573-12A>G variant since they were observed for both the wildtype and the mutant allele. Nevertheless, we aimed for an interpretation of these incidental findings. It has been shown previously that 0.8% of *RPGR* transcripts in the human retina demonstrate exon 12 skipping [38]. Intron 14 retention has been demonstrated in a minigene assay comprising exon 13, intron 13, exon 14, intron 14, and ORF15 [39]. Moreover, intron 14 retention has also been shown to occur in the human retinal pigment epithelium cell line ARPE-19 [39]. When we investigated RNAseq datasets derived from healthy human retina and brain donor samples, we saw evidence of both exon 12 skipping and intron 14 retention. Supplementary Figure S5 shows Sashimi plots of *RPGR* in two representative RNAseq datasets from human brain and retina. While exon 12 skipping is not evident in the retina sample (blue), the brain sample (red) shows a small number of reads linking exon 11 directly to exon 13. With respect to the retention of intron 14, both samples show a substantial number of reads mapping to intron 14. These results need to be interpreted with caution since intronic reads observed in RNAseq might stem from genomic DNA contamination in RNA preparations. However, at least in the brain sample, intronic reads that map to *RPGR* are sparse, consistent with a low level of genomic DNA contamination. The only exception is intron 14, to which a large number of reads map. This is highly indicative of intron 14 retention. Hence, the fact that our minigene assay for variant c.1573-12A>G revealed individual transcripts demonstrating exon 12 skipping and intron 14 retention might reflect physiological splicing events in the human brain (and possibly the retina).

## 4. Conclusions

We validated four NCSS variants in *RPGR* that are linked with XRP, thereby supporting a reclassification of these variants from VUS to likely pathogenic. These variants can now be implemented in routine diagnostic pipelines, thereby facilitating the genetic diagnosis in future RP patients. In addition, the re-classification of NCSS variants will open therapeutic opportunities for patients carrying these variants.

## 5. Materials and Methods

### 5.1. Patient Enrollment and Retrieval of Blood Samples

Five patients from four families were recruited and clinically examined at the Eye Hospital, University of Tübingen, Germany. Genomic DNA of patients was extracted from peripheral blood using standard protocols. Samples from all patients were recruited in accordance with the principles of the Declaration of Helsinki and were obtained with written informed consent accompanying the patients’ samples. The study was approved by the institutional review board of the Ethics Committee of the University Hospital of Tübingen under the study numbers 349/2003V and 116/2015BO2.

### 5.2. Clinical Evaluation

Patients underwent ophthalmic examination including detailed medical history, BCVA testing, kinetic perimetry (Octopus 900, Goldmann-III4e-Stimulus, Haag-Streit GmbH, Wedel, Germany), slit lamp examination, fundus examination and photography, and ERG. Color vision was examined in two patients by the Farnsworth D-15 test. In addition, several patients were tested using OCT and FAF (Spectralis^®^ OCT/HRA, 55 degrees, Heidelberg Engineering, Germany).

### 5.3. Diagnostic Testing

Sequence analysis of the *RPGR* gene was performed in two genetic diagnostic centers applying different analysis techniques. Patients XRP 29 and XRP 51 were screened by means of Sanger sequencing. Patients XRP 232 and XRP 235 underwent whole genome sequencing.

### 5.4. In Silico Analysis

The potential pathogenicity of the NCSS variants identified in this study was assessed with four different algorithms embedded in the Alamut visual software (v.2.12; Interactive Biosoftware, Rouen, France) using default settings.

RNA-seq data from healthy human retina and brain donors was aligned using hg19 as reference genome. BroadInstitute Integrated Genomics Viewer (IGV-2.3.40, Cambridge, MA, USA) was utilized to visualize aligned reads. Sashimi plots of *RPGR* were generated to visualize the distribution of intronic reads.

### 5.5. In Vitro Splice Assays

In vitro splice assays were performed as described previously [40]. Briefly, genomic segments encompassing the variant of interest along with flanking sequences were amplified from patient genomic DNA using a proofreading polymerase and cloned into the pSPL3 minigene plasmid vector. Cloned genomic segments were 695 bp for patient XRP 29 (GrCh37/hg19 X: 38,182,360-38,183,054; corresponding to exon 2 and flanking intronic sequences), 850 bp for XRP 51 (GrCh37/hg19 X: 38,181,662-38,182,511; corresponding to exon 3 and flanking intronic sequences), 999 bp for patient XRP 232 (GrCh37/hg19 X: 38,163,346-38,164,344; corresponding to exon 8 and flanking intronic sequences), and 4.8 kb for patient XRP 235 (GrCh37/hg19 X:g.38,146,653-38,151,496; corresponding to exon 12, intron 12, exon 13, intron 13, exon 14, and flanking intronic sequences), respectively. The wildtype alleles were derived from the respective mutant alleles by in vitro mutagenesis (IVM) using standard protocols. Following cloning, the resulting constructs in their wildtype and mutant versions were used to transfect HEK293T/17 cells (ATCC^®^ CRL-11268™), which were then analyzed with respect to splicing of minigene-derived transcripts using RT-PCR. Primers for PCR amplification, IVM, cDNA synthesis, and RT-PCR are available upon request. Genomic coordinates given in this manuscript are based on the GRCh37 genome (hg19). Mutation nomenclature is based on GenBank accession NM_001034853.1 with nucleotide one being the first nucleotide of the translation initiation codon ATG.

## Data Availability

The data that support the findings of this study are available from the Institute of Medical Genetics and Genomics (IMGAG) in Tübingen, and the Institute of Human Genetics in Munich, but restrictions apply to the availability of these data, which were used under license for the current study, and so are not publicly available. Data are however available from the authors upon reasonable request and with permission of the IMGAG and the Institute of Human Genetics in Munich.

## Supplementary Materials

Supplementary materials can be found at https://www.mdpi.com/1422-0067/22/2/850/s1. Figure S1: OCT scans of patients. Figure S2: Sequencing analysis of RT-PCR products obtained after transfection with the c.154+3_154+6del, c.247+5G>A, and c.779-5T>G minigene constructs. Figure S3: Sequencing analysis of RT-PCR products obtained after transfection with the c.1573-12A>G minigene constructs. Figure S4: Pedigrees of the patients analyzed in this study. Figure S5: Sashimi plots of *RPGR* obtained from RNAseq data from human brain and retina donor samples.

## Abbreviations

ACMG	American College of Medical Genetics and Genomics
ADRP	autosomal dominant retinitis pigmentosa
ARRP	autosomal recessive retinitis pigmentosa
BCVA	best corrected visual acuity
ERG	electroretinography
FAF	fundus autofluorescence
HEK	human embryonic kidney
HGMD	Human Gene Mutation Database
NCSS	non canonical splice site
OCT	optical coherence tomography
PDE6δ	phosphodiesterase 6 subunit delta
SRP	simplex/sporadic retinitis pigmentosa
RCC1	regulator of chromosome condensation 1
RP	retinitis pigmentosa
RPGR	retinitis pigmentosa GTPase regulator
RPGRIP1	RPGR interacting protein 1
RT-PCR	reverse transcription polymerase chain reaction
VUS	variant of uncertain significance
XRP	X-linked retinitis pigmentosa
