# Supplementary material for: X-Linked Retinitis Pigmentosa Caused by Non-Canonical Splice Site Variants in RPGR
Source: Int J Mol Sci. 2021 Jan 16;22(2):850. doi: 10.3390/ijms22020850 (PMC7830253; doi:10.3390/ijms22020850)
Supplement: Supplementary file 1 [file ijms-22-00850-s001.zip › ijms-1029900-supplementary/Supplementary Figure S1.docx]

**Supplementary Figure S1:** OCT scans of patients.
