# Supplementary material for: X-Linked Retinitis Pigmentosa Caused by Non-Canonical Splice Site Variants in RPGR
Source: Int J Mol Sci. 2021 Jan 16;22(2):850. doi: 10.3390/ijms22020850 (PMC7830253; doi:10.3390/ijms22020850)
Supplement: Supplementary file 1 [file ijms-22-00850-s001.zip › ijms-1029900-supplementary/Supplementary Figure S2.docx]

**c.154+3_154+6del wildtype construct**

**c.154+3_154+6del mutant construct**

**
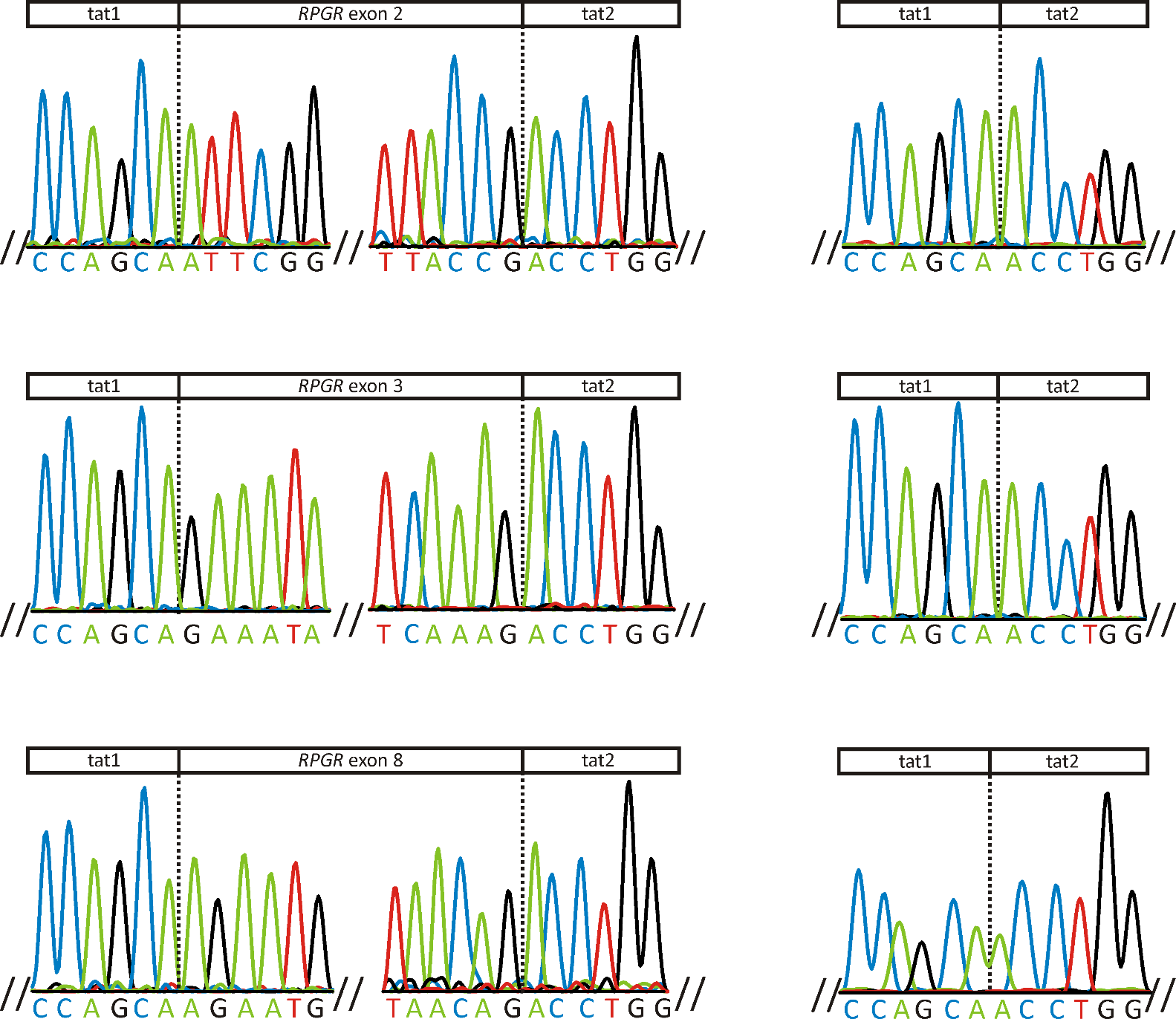
**

**c.247+5G>A mutant construct**

**c.247+5G>A wildtype construct**

**c.779-5T>G mutant construct**

**c.779-5T>G wildtype construct**

**Supplementary Figure S2:** Sequencing analysis of RT-PCR products obtained after transfection with the c.154+3_154+6del, c.247+5G>A, and c.779-5T>G minigene constructs. Sequencing analysis shows that the RT-PCR products derived from transfection with the respective wildtype minigene constructs correspond to correct splicing (i.e. splicing of the respective *RPGR* exon between the pSPL3 exons tat1 and tat2) while the transcripts from the mutant minigene constructs show skipping of the respective exon (i.e. tat1 and tat2 are spliced directly adjacent to each other).
