# Supplementary material for: X-Linked Retinitis Pigmentosa Caused by Non-Canonical Splice Site Variants in RPGR
Source: Int J Mol Sci. 2021 Jan 16;22(2):850. doi: 10.3390/ijms22020850 (PMC7830253; doi:10.3390/ijms22020850)
Supplement: Supplementary file 1 [file ijms-22-00850-s001.zip › ijms-1029900-supplementary/Supplementary Figure S3.docx]

**
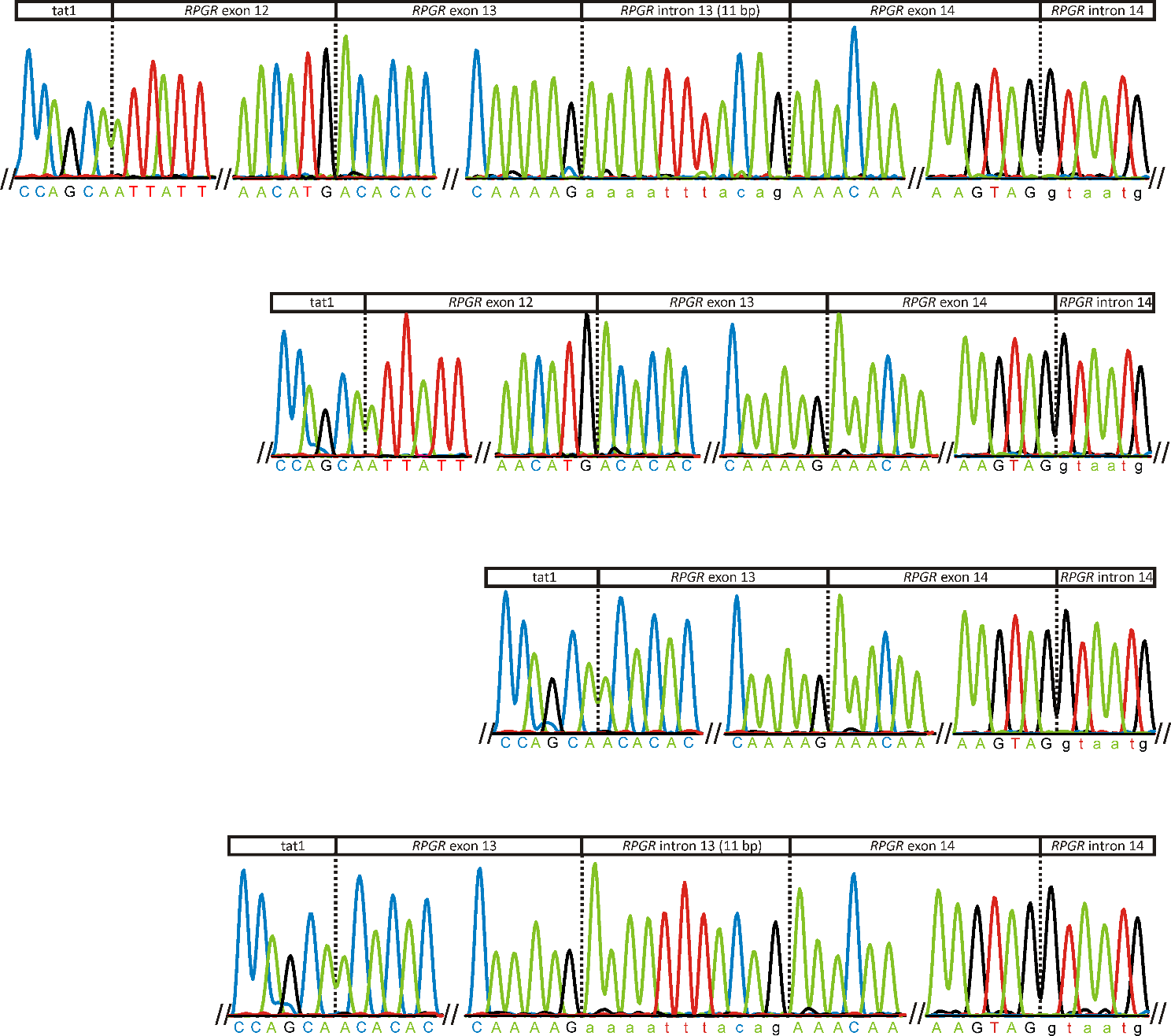
**

**c.1573-12A>G wildtype construct**

**RT-PCR product E**

**c.1573-12A>G mutant construct**

**RT-PCR product G**

**c.1573-12A>G wildtype construct**

**RT-PCR product F**

**c.1573-12A>G mutant construct**

**RT-PCR product H**

**Supplementary Figure S3:** Sequencing analysis of subcloned RT-PCR products obtained after transfection with the c.1573-12A>G minigene constructs.

The bigger RT-PCR product derived from transfection with the wildtype minigene construct (band labeled with E in Figure 3) corresponds to correct splicing of *RPGR* exon 13 between exons 12 and 14, while the bigger product derived from transfection with the mutant minigene construct construct shows inclusion of the last eleven nucleotides of intron 13 (band G in Figure 3). The smaller RT-PCR products (bands F and H in Figure 3) seen after transfection with both the wildtype and the mutant minigene construct result from exon 12 skipping. Note that all RT-PCR products show retention of intron 14. The junction of intron 14 and the pSPL3 exon tat2 is not depicted.
