# Supplementary material for: X-Linked Retinitis Pigmentosa Caused by Non-Canonical Splice Site Variants in RPGR
Source: Int J Mol Sci. 2021 Jan 16;22(2):850. doi: 10.3390/ijms22020850 (PMC7830253; doi:10.3390/ijms22020850)
Supplement: Supplementary file 1 [file ijms-22-00850-s001.zip › ijms-1029900-supplementary/Supplementary Figure S4.docx]

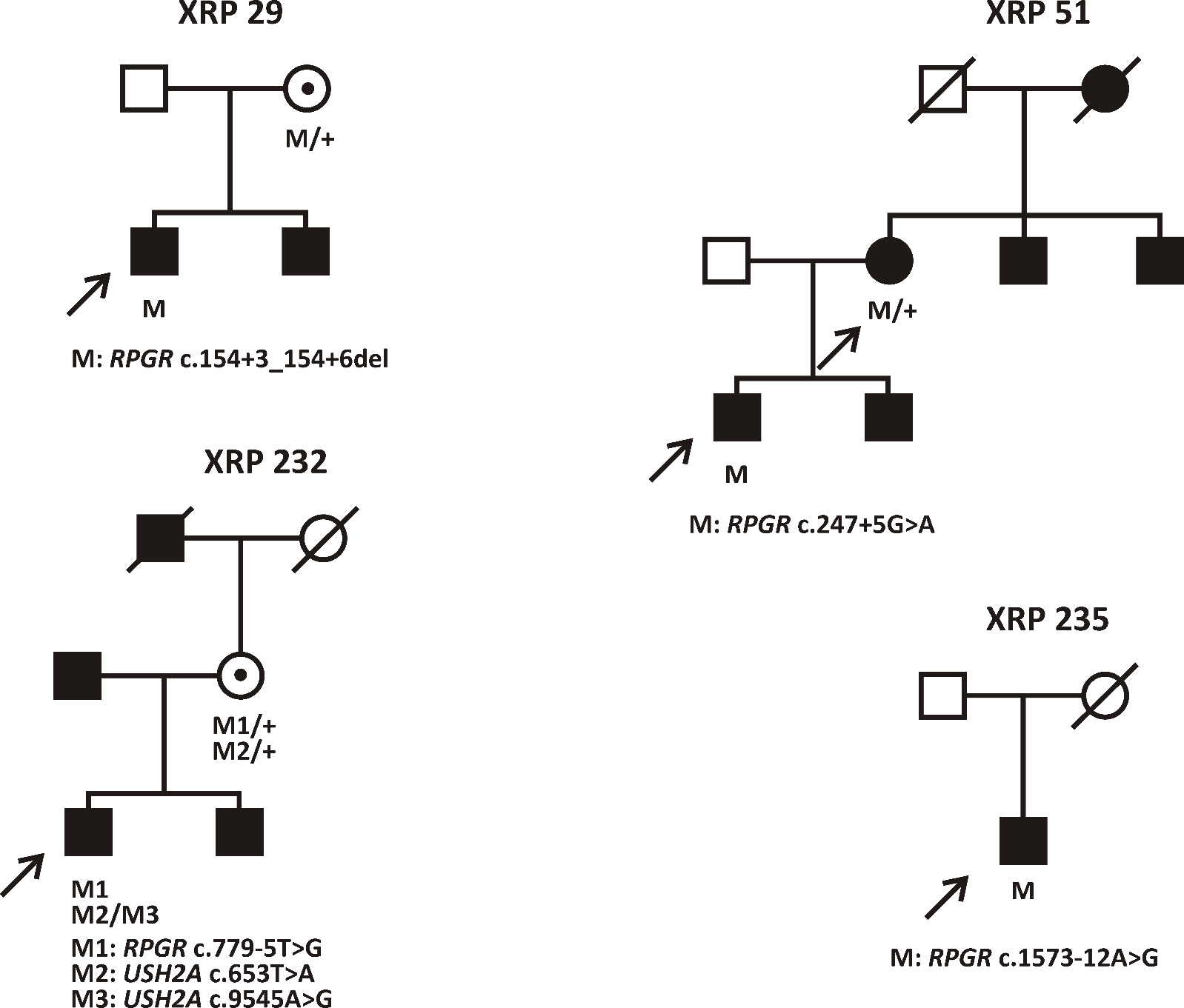


**Supplementary Figure S4:** Pedigrees of the patients analyzed in this study.

Genotypes are given below each available family member. M, mutant allele; +, wildtype allele. Patients from whom clinical data were available (see Figure 1) are indicated by an arrow. Note that the presence of carrier phenotypes in the patients´ mothers has not been validated with the exception of XRP 51.
