# Supplementary material for: X-Linked Retinitis Pigmentosa Caused by Non-Canonical Splice Site Variants in RPGR
Source: Int J Mol Sci. 2021 Jan 16;22(2):850. doi: 10.3390/ijms22020850 (PMC7830253; doi:10.3390/ijms22020850)
Supplement: Supplementary file 1 [file ijms-22-00850-s001.zip › ijms-1029900-supplementary/Supplementary Figure S5.docx]

**
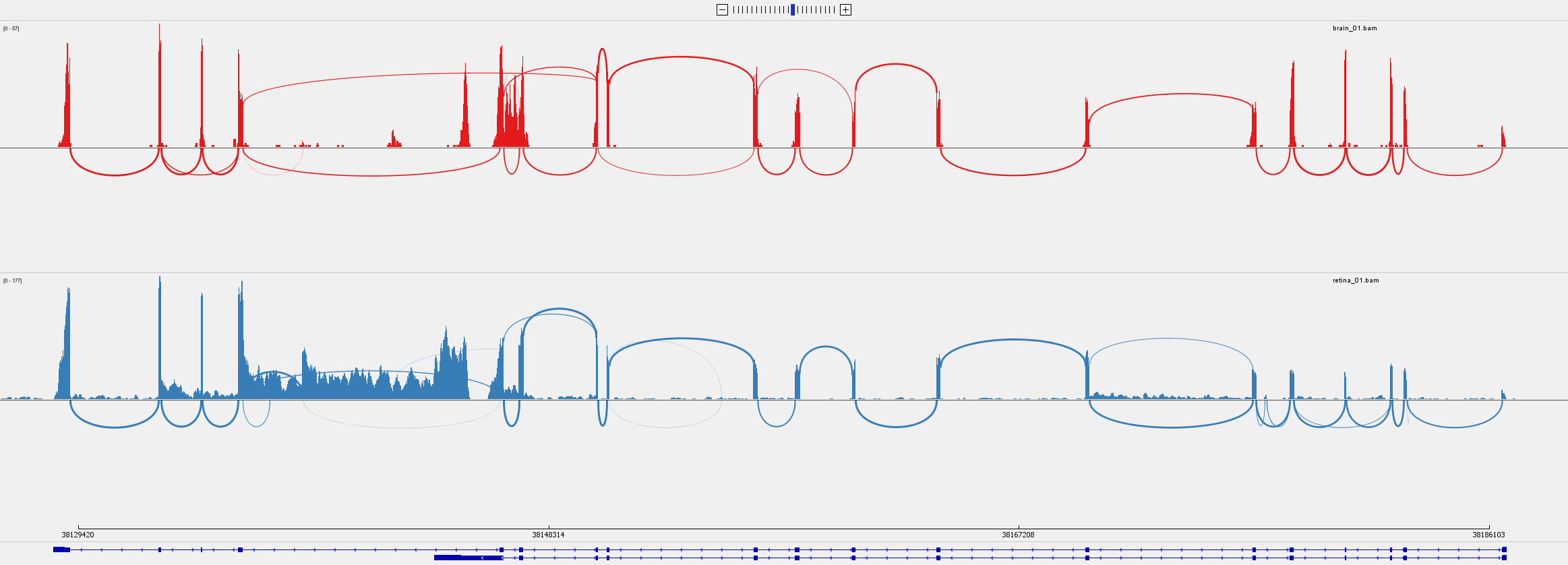
**

***RPGR*^Ex1-19^**

***RPGR*^ORF15^**

**^Ex11^**

**^Ex13^**

**^Ex12^**

**Supplementary Figure S5:** Sashimi plots of *RPGR* obtained from RNAseq data from human brain and retina donor samples. Two representative datasets from brain (red) and retina (blue) are shown. Vertical bars indicate exonic reads. The arcs between exonic reads indicate splice junction reads. The two isoforms of *RPGR* are shown below the Sashimi plots with blue vertical bars indicating the exons. Intron 14 of the *RPGR*^ORF15^ isoform is marked by a black rectangle. The comparably high number of reads mapping to intron 14 in the brain dataset is indicative of intron 14 retention. The brain sample also shows a small number of reads (marked by black arrow) linking exon 11 directly to exon 13, indicating exon 12 skipping.
